# Vegemite Beer: yeast extract spreads as nutrient supplements to promote fermentation

**DOI:** 10.7717/peerj.2271

**Published:** 2016-08-10

**Authors:** Edward D. Kerr, Benjamin L. Schulz

**Affiliations:** School of Chemistry and Molecular Biosciences, The University of Queensland, Brisbane, Queensland, Australia

**Keywords:** Yeast, Vegemite, Fermentation, Ethanol, Yeast extract

## Abstract

Vegemite is an iconic Australian food spread made from spent brewers’ yeast extract, which has been reported to be used as an ingredient in illegal home brewing. In this study, we tested the utility of Vegemite and the similar spread Marmite in promoting fermentation. We could not culture microorganisms from either Vegemite or Marmite, consistent with these food-grade spreads being essentially sterile. To test if the addition of Vegemite or Marmite could assist in fermentation when additional viable yeast was also present, solutions containing glucose and a range of concentrations of either Vegemite or Marmite were inoculated with brewers’ yeast. No fermentation occurred in any condition without addition of extra brewer’s yeast. Fermentation did not occur when yeast was inoculated into solutions containing only glucose, but progressed efficiently with when Vegemite or Marmite was also added. Gas Chromatography confirmed that ethanol was present at ∼3% v/v post-fermentation in all samples which contained glucose, Vegemite or Marmite, and brewers’ yeast. Trace amounts of methanol were also detected. Mass spectrometry proteomics identified abundant intracellular yeast proteins and barley proteins in Vegemite and Marmite, and abundant secreted yeast proteins from actively growing yeast in those samples to which extra brewers’ yeast had been added. We estimate that the real-world cost of home brewed “Vegemite Beer” would be very low. Our results show that Vegemite or other yeast extract spreads could provide cheap and readily available sources of nutrient supplementation to increase the efficiency of fermentation in home brewing or other settings.

## Introduction

Vegemite is an iconic Australian food spread made from brewers’ yeast extract. Although very popular in Australia and internationally as a spread on bread or toast, and as an ingredient in other foods, Vegemite has been recently reported to be used as a source of yeast for home brewing in indigenous Australian communities in which alcohol is banned (Cormack, 2015). Vegemite is also banned in prisons in Victoria, Australia, due to its reported use in home brew alcohol production (Sexton, 2007). As the Vegemite production process would be expected to effectively sterilize the spread, it is unclear how addition of Vegemite would benefit alcohol production.

The yeast *Saccharomyces cerevisiae* can be grown on a variety of rich or synthetic defined media. To allow yeast growth these media must contain a nitrogen source, a carbon source, and additional nutrients or vitamins. The most common rich medium used is YPD, containing yeast extract, peptone, and dextrose (Sherman, 1991). Common derivatives of this medium exchange dextrose for an alternative carbon source such as galactose, glycerol, or ethanol. Defined minimal synthetic media typically contains yeast nitrogen base, defined amino acids, and a carbon source, most commonly dextrose (Styles, 2001). Synthetic media can easily be made lacking one or more component (e.g., uracil, leucine, histidine, methionine) for maintenance of plasmids or selection of genomic integration events in yeast strains with defined auxotrophies.

Vegemite and Marmite are very similar food spreads made from breweries’ spent yeast. Vegemite is an Australian product, while Marmite is made by English and New Zealand companies. Both Vegemite and Marmite are food pastes made from autolysed yeast extract. Spent brewing yeast are autolyzed by the addition of sodium chloride, and heated to sterilize and concentrate the paste.

Both Vegemite and Marmite have been previously used as ingredients in microbiological growth media. Vegemite has been used in storage media for *S. cerevisiae* (Orlowski & Barford, 1987), and Marmite has been included in growth media for a range of bacteria including *Micrococcus* and *Streptococcus* (Kelly, Miller & Hale, 1952; Marquis, Brown & Fenn, 1971). In these reports, Vegemite or Marmite were used in media in place of autolysed yeast extract (Kelly, Miller & Hale, 1952).

In this study, we investigated the utility of Vegemite and Marmite in promoting fermentation and yeast growth. We tested both spreads for the presence of culturable microorganisms, investigated the dose-dependent response of *S. cerevisiae* to growth in liquid media with varying concentrations of either spread, and performed gas chromatography and mass spectrometry proteomic analyses of fermentation end products with addition of Vegemite or Marmite.

## Materials & Methods

### Fermentation

Jars of Vegemite and Marmite were purchased from three independent retail outlets in Brisbane, Australia. Spread material was streaked on Yeast Peptone Dextrose (YPD) agar plates directly or after 10-fold dilution in sterile water and incubated at 25 °C for 7 days.

**Figure 1:**
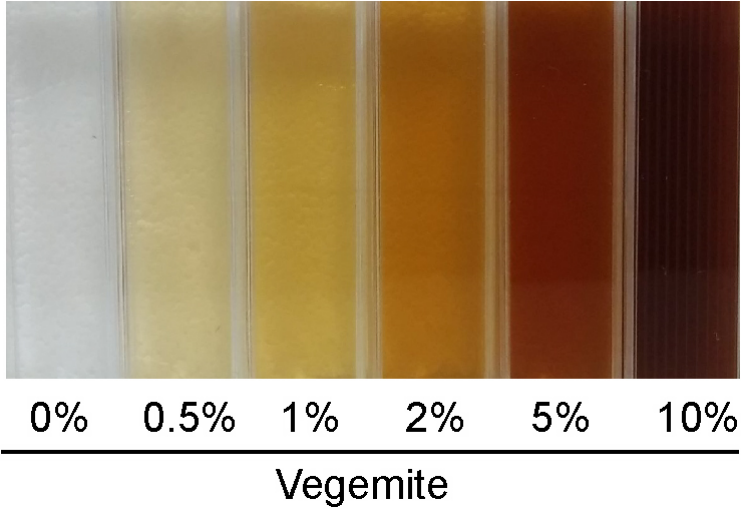
Solutions of glucose and various concentrations of Vegemite (0%, 0.5%, 1%, 2%, 5%, and 10%, all v/v).

Fermentations were performed in 30 mL of liquid media in sterile 50 mL Falcon tubes. Solutions of 8% v/v glucose and 20% v/v Vegemite or Marmite in water were separately sterilized by autoclave, and mixed to give media with final concentrations of 4% v/v glucose with 0% v/v, 0.5% v/v, 1% v/v, 2% v/v, 5% v/v or 10% v/v of either Vegemite or Marmite, and additional sterile water to give a total volume of 30 mL. Vegemite and Marmite were largely soluble at these concentrations, with only small amounts of insoluble material rapidly sedimenting (Fig. 1). Media was either inoculated or not with 10 µL of brewers’ yeast (Mangrove Jacks, Craft series M79, Burton Union) pre-grown to saturation in YPD. The weight of each sample was measured daily for eight days to follow the course of fermentation.

### Gas chromatography

1 mL of each post-fermentation media sample was combined with 200 µL of 5 M NaCl prior to analysis. Standard solutions of ethanol, acetaldehyde, formaldehyde, and methanol were made to the same volume and NaCl concentration. Gas chromatography (GC) headspace analysis was performed using a Clarus 600 with a Turbomatrix 110 Headspace sampler (PerkinElmer). Headspace sampling was performed with an oven temperature of 70 °C, injection time of 0.04 min, and column pressure of 16 psi, Samples were separated using a Restek Rxi-624Sil Column (30 m length, 0.32 mm ID) with He as a carrier gas at 2.5 mL/min at 90 °C, and a GC cycle time of 3.2 min.

### Mass spectrometry proteomics

Proteins from 10 µL of post-fermentation media were precipitated as described (Xu et al., 2014) by addition of 100 µL 1:1 methanol/acetone, incubation at −20 °C for 16 h, and centrifugation at 16,000 rcf for 10 min. Proteins were resuspended in 100 µL of 100 mM ammonium acetate, 10 mM dithiothreitol with 1 µg trypsin (Proteomics grade; Sigma), digested at 37 °C with shaking for 16 h, and desalted with C18 ZipTips. Peptides were detected by liquid chromatography electrospray ionization tandem mass spectrometry (LC-MS/MS) using a Prominence nanoLC system (Shimadzu) and TripleTof 5600 mass spectrometer with a Nanospray III interface (SCIEX) essentially as previously described (Xu, Bailey & Schulz, 2015). Peptides were separated with buffer A (1% acetonitrile v/v and 0.1% formic acid v/v) and buffer B (80% acetonitrile v/v with 0.1% formic acid v/v) with a gradient of 10–60% buffer B over 15 min. Gas and voltage setting were adjusted as required. Information-dependent acquisition (IDA) was performed, with an MS-TOF scan from an *m/z* of 350–1,800 for 0.5 s followed by IDA MS/MS with automated selection of the top 20 peptides from an *m/z* of 40–1,800 for 0.05 s per spectrum. Collision energy was automatically assigned for each peptide by the Analyst software (SCIEX). Peptide identification was performed essentially as previously described (Bailey, Jamaluddin & Schulz, 2012) using ProteinPilot 4.1 (SCIEX), searching the UniProt database (downloaded from http://uniprot.org on 15 January 2015; 16 818 973 sequences) with standard settings: sample type, identification; cysteine alkylation, none; instrument, TripleTof 5600; species, none; ID focus, biological modifications; enzyme, trypsin; Search effort, thorough ID. False discovery rate analysis using ProteinPilot was performed on all searches with limits of 99% identification confidence and 1% local false discovery rate. Common contaminants were removed from search results. Mass spectrometry data have been deposited to the ProteomeXchange Consortium (http://proteomecentral.proteomexchange.org) (Vizcaíno et al., 2014) via the PRIDE partner repository with the dataset identifier PXD003402.

**Figure 2:**
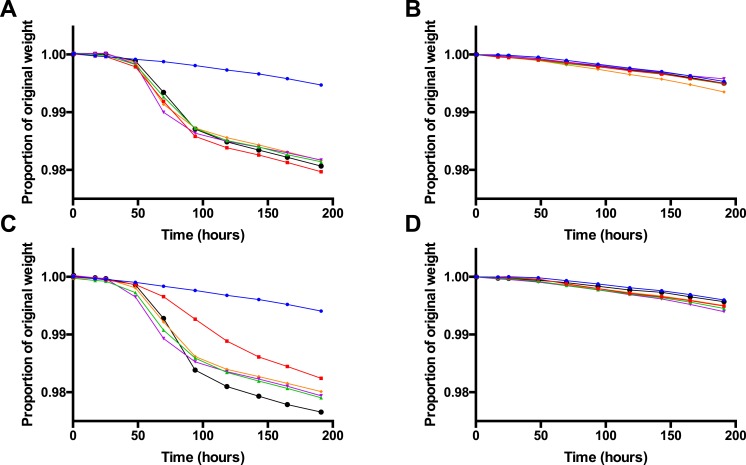
Fermentation kinetics in the presence of glucose, Vegemite or Marmite, and yeast. Proportion of original sample weight over the course of incubation in solutions with 4% glucose and varying concentrations (0% blue, 0.5% red, 1% green, 2% purple, 5% orange, or 10% black, all v/v) of Vegemite (A, B) or Marmite (C, D), with (A, C) or without (B, D) inoculation with additional viable brewers’ yeast.

**Figure 3:**
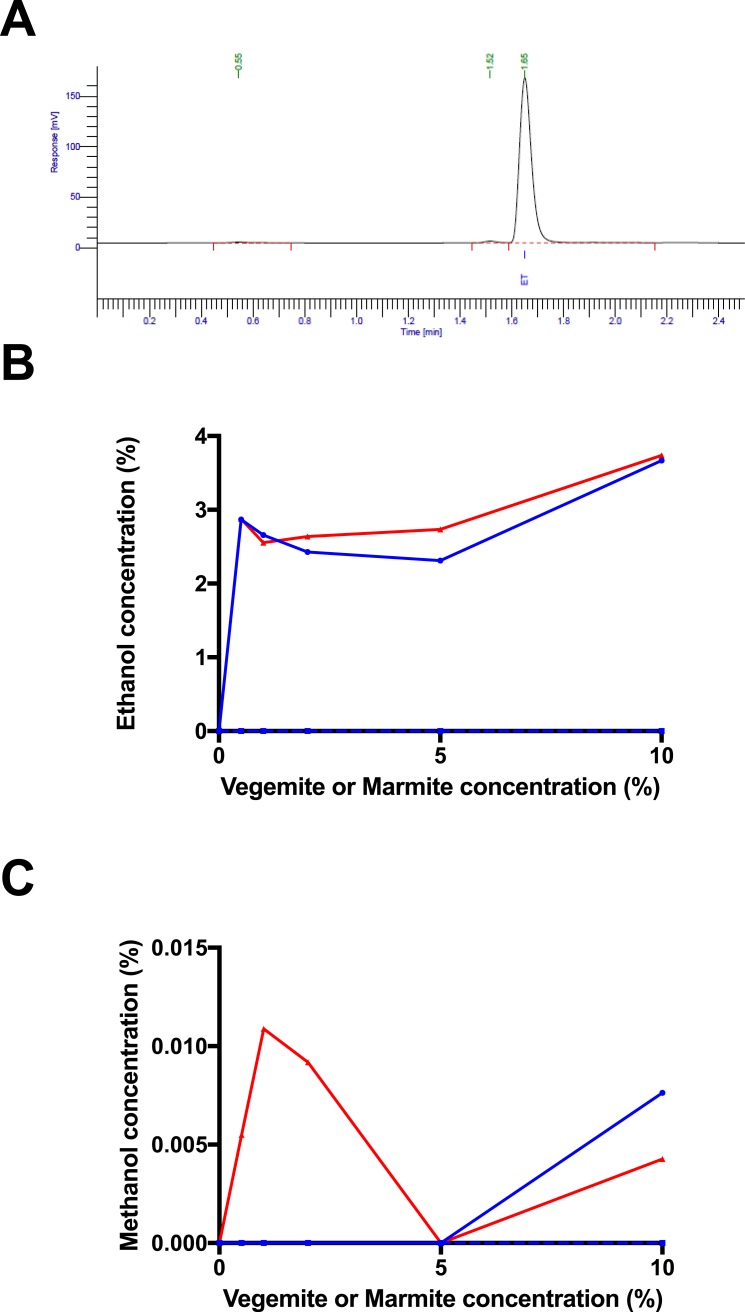
Post-fermentation headspace GC analysis. (A) Headspace GC trace for post-fermentation sample with 4% glucose, 1% Vegemite, and brewers’ yeast. Peaks correspond to an unknown compound at 0.54 min, methanol at 1.52 min, and ethanol at 1.65 min. Post-fermentation (B) ethanol and (C) methanol concentrations in samples with 4% glucose, varying concentrations of Vegemite (red) or Marmite (blue) with (full) or without (dashed) inoculation with additional viable brewers’ yeast.

## Results

### Fermentation

To test if Vegemite and Marmite purchased from retail outlets contained viable microorganisms, we streaked material from both spreads at two dilutions on YPD agar plates and incubated them for 7 days at 25 °C. No growth was observed at any condition from Vegemite or Marmite, indicating that no microorganisms were culturable from either spread.

As yeast was not culturable from Vegemite or Marmite, we next investigated if either spread could be used as a nutrient source to promote the growth of *S. cerevisiae*. A commercial home brewing strain of brewers’ yeast was inoculated in liquid media containing 4% v/v glucose and various concentrations of Vegemite or Marmite ranging from 0–10% v/v. The extent of fermentation was monitored by measuring the weight of each sample daily over 8 days, as fermentation of glucose to ethanol and CO_2_ results in weight loss from degasification of dissolved CO_2_. Only minimal weight loss consistent with evaporation of water was observed in samples to which no yeast had been inoculated (Figs. 2B and 2D). Similarly, no weight loss was observed in samples with only glucose and yeast (Figs. 2A and 2C). However, addition of glucose, yeast, and any amount of either Vegemite or Marmite resulted in substantial weight loss between days 2 and 4 (Figs. 2A and 2C). Extensive effervescence was also observed in the samples that lost weight over this period, consistent with CO_2_ production from active fermentation. Addition of Vegemite at any concentration from 0.5 –10% v/v resulted in equivalent weight loss kinetics (Fig. 2A). In contrast, addition of 0.5% v/v Marmite resulted in slower weight loss, and 10% v/v Marmite faster weight loss, than 1–5% v/v Marmite (Fig. 2C).

**Table 1:** Proteins identified by LC-MS/MS post-fermentation with 4% glucose and 2% Vegemite without inoculation of additional viable yeast.

Protein name	Accession	Peptides (95%)	%Cov (95)	Score
Enolase 1 (Yeast)	P00924	14	28.60	24
Glyceraldehyde-3-phosphate dehydrogenase 1 (Yeast)	P00360	8	23.49	16
Phosphoglycerate kinase (Yeast)	P00560	7	20.43	14
Pyruvate kinase 1 (Yeast)	P00549	6	14.00	12.27
Serpin-Z4 (Barley)	P06293	6	20.05	12
Alcohol dehydrogenase 1 (Yeast)	P00330	6	22.41	11.72
Fructose-bisphosphate aldolase (Yeast)	P14540	5	21.17	10
Alpha-amylase/trypsin inhibitor CMd (Barley)	P11643	5	50.88	10
Phosphoglycerate mutase 1 (Yeast)	P00950	3	12.96	6.06
Glyceraldehyde-3-phosphate dehydrogenase 2 (Yeast)	P00358	5	17.17	6
Elongation factor 1-alpha (Yeast)	P02994	3	9.39	6
Lysophospholipase 2 (Yeast)	Q03674	2	2.98	4
Cell wall mannoprotein PIR1 (Yeast)	Q03178	3	5.28	4
Alpha-amylase/trypsin inhibitor CMb (Barley)	P32936	2	12.75	4
Non-specific lipid-transfer protein 1 (Barley)	P07597	2	21.37	4
Heat shock protein SSA1 (Yeast)	P10591	2	3.74	2.91
Enolase 2 (Yeast)	P00925	12	21.97	2
Glyceraldehyde-3-phosphate dehydrogenase 3 (Yeast)	P00359	6	20.48	2

**Table 2:** Proteins identified by LC-MS/MS post-fermentation with 4% glucose and 2% Vegemite with inoculation of additional viable yeast.

Name	Accession	Peptides (95%)	%Cov (95)	Score
Probable family 17 glucosidase SCW4 (Yeast)	P53334	15	39.64	28.8
Enolase 1 (Yeast)	P00924	13	40.05	24.15
Glyceraldehyde-3-phosphate dehydrogenase 2 (Yeast)	P00358	12	37.05	22.25
Phosphoglycerate kinase (Yeast)	P00560	7	23.56	14
Fructose-bisphosphate aldolase (Yeast)	P14540	6	15.88	11.99
Alcohol dehydrogenase 1 (Yeast)	P00330	6	20.11	11.72
Elongation factor 1-alpha (Yeast)	P02994	5	15.50	10
Probable secreted beta-glucosidase UTH1 (Yeast)	P36135	5	19.18	9.27
Cell wall mannoprotein PST1 (Yeast)	Q12355	6	15.99	7.44
Probable secreted beta-glucosidase SIM1 (Yeast)	P40472	6	17.23	6.08
Glyceraldehyde-3-phosphate dehydrogenase 1 (Yeast)	P00360	10	31.63	6.05
Phosphoglycerate mutase 1 (Yeast)	P00950	4	15.38	5.91
Probable family 17 glucosidase SCW10 (Yeast)	Q04951	3	12.60	5.82
Pyruvate kinase 1 (Yeast)	P00549	3	5.60	4.24
Enolase 2 (Yeast)	P00925	13	30.21	4.02
Cell wall mannoprotein CIS3 (Yeast)	P47001	2	7.93	4
Protein YGP1 (Yeast)	P38616	2	8.47	4
Cell wall protein ECM33 (Yeast)	P38248	2	6.99	4
Alpha-amylase/trypsin inhibitor CMb (Barley)	P32936	2	14.77	3.85
Non-specific lipid-transfer protein 1 (Barley)	P07597	2	21.37	2.68
Cell wall mannoprotein HSP150 (Yeast)	P32478	2	4.36	2

**Table 3:** Proteins identified by LC-MS/MS post-fermentation with 4% glucose and 2% Marmite without inoculation of additional viable yeast.

Name	Accession	Peptides (95%)	%Cov (95)	Score
Enolase 1 (Yeast)	P00924	24	49.20	42.58
Glyceraldehyde-3-phosphate dehydrogenase 3 (Yeast)	P00359	31	47.89	36.18
Glyceraldehyde-3-phosphate dehydrogenase 1 (Yeast)	P00360	38	47.59	19.79
Phosphoglycerate kinase (Yeast)	P00560	8	24.76	18.2
Fructose-bisphosphate aldolase (Yeast)	P14540	11	27.86	17.7
Heat shock protein SSA1 (Yeast)	P10591	12	19.31	16.64
Pyruvate kinase 1 (Yeast)	P00549	8	12.20	13.73
Elongation factor 1-alpha (Yeast)	P02994	11	19.87	12.99
Phosphoglycerate mutase 1 (Yeast)	P00950	8	26.72	12.25
Heat shock protein 26 (Yeast)	P15992	9	35.51	11.99
Alpha-amylase/trypsin inhibitor CMd (Barley)	P11643	5	49.71	9.74
Alcohol dehydrogenase 1 (Yeast)	P00330	7	23.56	9.22
Enolase 2 (Yeast)	P00925	23	40.05	6.17
Pyruvate decarboxylase isozyme 1 (Yeast)	P06169	6	8.53	4.9
Protein YGP1(Yeast)	P38616	3	12.99	4.34
Plasma membrane ATPase 2 (Yeast)	P19657	2	2.53	2
Barwin (Barley)	P28814	2	20.80	4
40S ribosomal protein S5 (Yeast)	P26783	2	12.44	4
ATP synthase subunit beta, mitochondrial (Yeast)	P00830	2	5.09	3.92
Triosephosphate isomerase (Yeast)	P00942	3	12.10	3.65
Alpha-amylase/trypsin inhibitor CMb (Barley)	P32936	2	18.12	3.4
Serpin-Z2A (Barley)	Q9ST57	2	7.79	3.14
40S ribosomal protein S3 (Yeast)	P05750	2	9.58	3.12
60S ribosomal protein L38 (Yeast)	P49167	2	28.21	2.84
Elongation factor 2 (Yeast)	P32324	2	2.61	2.41
14-3-3-like protein A (Barley)	P29305	2	3.82	2
Aconitate hydratase, mitochondrial (Yeast)	P19414	3	4.88	2.27
Thioredoxin-2 (Yeast)	P22803	2	12.50	2.14

**Table 4:** Proteins identified by LC-MS/MS post-fermentation with 4% glucose and 2% Marmite with inoculation of additional viable yeast.

Name	Accession	Peptides (95%)	%Cov (95)	Score
Enolase 1 (Yeast)	P00924	16	35.70	29.05
Glyceraldehyde-3-phosphate dehydrogenase 1 (Yeast)	P00360	16	39.16	24.35
Probable family 17 glucosidase SCW4 (Yeast)	P53334	11	29.53	22.6
Phosphoglycerate kinase (Yeast)	P00560	9	25.48	16.7
Glyceraldehyde-3-phosphate dehydrogenase 2 (Yeast)	P00358	10	33.73	10.04
Heat shock protein SSA1 (Yeast)	P10591	6	10.12	10.01
Elongation factor 1-alpha (Yeast)	P02994	6	19.65	9.84
Glucan 1,3-beta-glucosidase I/II (Yeast)	P23776	5	13.17	9.66
Cell wall mannoprotein PST1 (Yeast)	Q12355	5	15.77	8.92
Alcohol dehydrogenase 1 (Yeast)	P00330	4	13.22	8.39
Pyruvate kinase 1 (Yeast)	P00549	4	9.00	8.05
Enolase 2 (Yeast)	P00925	15	30.43	8
Phosphoglycerate mutase 1 (Yeast)	P00950	4	20.24	7.22
Heat shock protein 26 (Yeast)	P15992	3	21.50	6.32
Protein YGP1 (Yeast)	P38616	3	12.99	6
Fructose-bisphosphate aldolase (Yeast)	P14540	3	15.88	6
Cell wall protein ECM33 (Yeast)	P38248	3	9.32	5.89
Glyceraldehyde-3-phosphate dehydrogenase 3 (Yeast)	P00359	11	36.75	4
40S ribosomal protein S14-B (Yeast)	P39516	2	9.42	2
40S ribosomal protein S3 (Yeast)	P05750	2	9.58	3.68
Probable secreted beta-glucosidase UTH1 (Yeast)	P36135	2	9.04	3.55
Triosephosphate isomerase (Yeast)	P00942	2	10.08	3.26
40S ribosomal protein S19-B (Yeast)	P07281	2	13.89	2.57
Cell wall mannoprotein HSP150 (Yeast)	P32478	2	2.42	2.49
Seripauperin-9 (Yeast)	Q3E770	2	11.67	2

**Figure 4:**
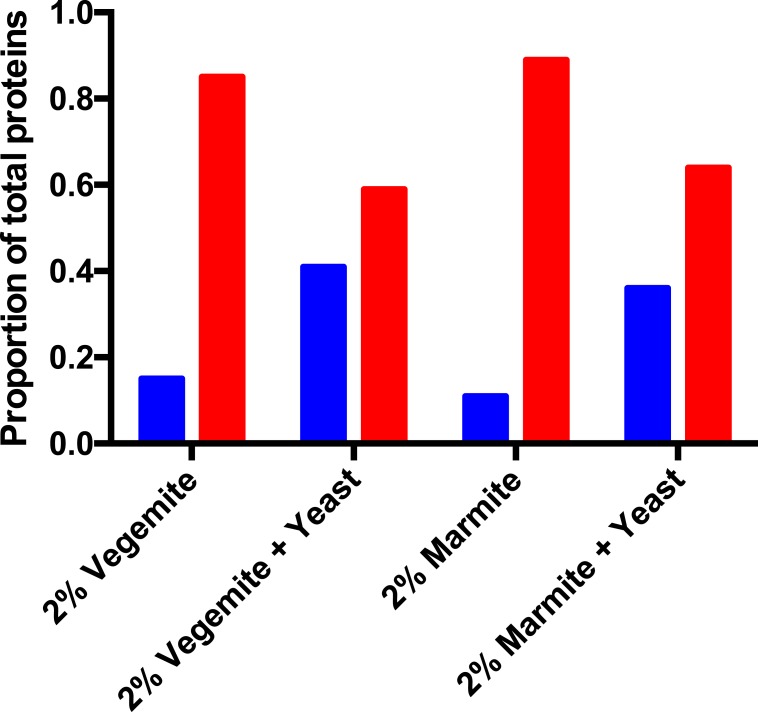
Post-fermentation mass spectrometry proteomics. Proportion of secreted (blue) and intracellular (red) yeast proteins in post-fermentation samples with 4% glucose, and 2% Vegemite or Marmite, with or without inoculation with additional viable brewers’ yeast.

### Gas chromatography

To confirm that fermentation had occurred in samples incubated with glucose, yeast, and either Vegemite or Marmite, we performed headspace GC analysis to quantify the amount of ethanol in each post-fermentation sample (peak at 1.65 min in Fig. 3A). Headspace GC detected ethanol at ∼3% v/v in all samples incubated with glucose, yeast, and either Vegemite or Marmite (Fig. 3B). No ethanol was detected in samples with only glucose and yeast, or with only glucose and either Vegemite or Marmite. These results are consistent with our measurements of weight loss over the course of fermentation (Fig. 2), and indicate that effective fermentation of glucose to ethanol can be achieved with media containing glucose, either Vegemite or Marmite, and additional viable yeast.

Trace amounts of other components were also detected by headspace GC analysis. The additional major trace compound was identified as methanol by co-elution with a standard (peak at 1.52 min in Fig. 3A), and was present at very low amounts up to ∼0.01% v/v in samples incubated with glucose, yeast, and either Vegemite or Marmite (Fig. 3C). We were not able to identify the additional minor trace compound (peak at 0.55 min in Fig. 3A).

### Mass spectrometry proteomics

We performed qualitative mass spectrometry proteomics to provide additional validation that inclusion of Vegemite or Marmite to glucose solutions promoted the growth of yeast. We precipitated proteins in post-fermentation samples, digested them with trypsin, detected peptides with LC-MS/MS, and identified peptides and proteins in each sample with database searching. We identified proteins present in samples with 4% v/v glucose, and either 2% v/v Vegemite or Marmite, with or without inoculation with brewers’ yeast. Samples not inoculated with additional yeast contained diverse proteins from both yeast and barley, consistent with the ingredients and production process of Vegemite and Marmite. Post-fermentation samples with glucose, Vegemite or Marmite, and additional yeast similarly contained proteins from yeast and barley, but with qualitative differences. The major categories of yeast proteins identified in all samples were secreted cell wall mannoproteins, ribosomal proteins, heat shock proteins, and enzymes involved in central carbon metabolism (Tables 1–4). Gene Ontology (GO) term analysis revealed that samples to which additional brewers’ yeast had been inoculated had more secreted proteins (GO:0005576 Extracellular) than samples with only Vegemite and Marmite, compared with the number of intracellular proteins (Fig. 4). This is consistent with secretion of proteins from actively growing yeast in media containing glucose, yeast, and Vegemite or Marmite.

## Discussion

We have investigated the mechanisms by which yeast extract spreads such as Vegemite may be of use in promoting ethanol production. We could not culture microorganisms from Vegemite or Marmite purchased from retail outlets, suggesting that there is no viable yeast in these spreads which could initiate fermentation. However, fermentation proceeded efficiently when we incubated solutions of glucose, and either Vegemite or Marmite, with additional viable brewers’ yeast. We measured the extent of fermentation by weight loss during incubation, and confirmed fermentative ethanol production with headspace GC analysis. Qualitative proteomics identified abundant secreted yeast proteins in post-fermentation samples, additionally consistent with active growth of yeast. It is most likely that Vegemite and Marmite provide a rich nitrogen and nutrient source for the inoculated yeast, equivalent to autolysed yeast extract in standard laboratory growth media.

The rate and extent of fermentation was highly robust to the concentration of Vegemite or Marmite, with all samples completing fermentation within 3–4 days, and reaching a final ethanol concentration of ∼3% v/v. No inhibitory effects were observed at the highest Vegemite or Marmite concentrations tested. Vegemite and Marmite are known to be high in salt, which acts as a preservative by inhibiting the growth of many microorganisms, including yeast. Vegemite contains 3% v/v NaCl, equivalent to a final concentration of 0.3% v/v NaCl (∼50 mM) at the highest concentration of Vegemite tested. This modest concentration is too low to affect *S. cerevisiae* growth.

The presence of trace amounts of methanol after fermentation was unexpected, but occurred at very low concentrations (Fig. 4). Although dependent on inoculation with brewers’ yeast, the origin of this methanol is unclear.

**Table 5:** Estimated comparative costs of “Vegemite beer”, home brew extract kit beer, and two common Australian commercial beers at retail.

Stage	Ingredient	Amount	Cost	Rationale
**“Vegemite Beer” (23 L) (∼5% v/v ethanol)**				
**Fermentation**	Water	23 L	$0.00	Cost negligible
	Sucrose	3 kg	$2.70	$1.80 per 2 kg (Coles)
	Vegemite	115 g	$1.81	$8.79 per 560 g jar (Coles)
	Yeast	35 g	$0.58	$4.62 per 280 g dried yeast (Coles)
**Bottling**	Sucrose	184 g	$0.17	8 g/L using sucrose
	Bottles		$0.00	Assume collected not bought
		**Cost per 23 L**	**$5.26**	
		**Cost per 375 mL equivalent**	**$0.09**	
**Retail Homebrew kit (23 L) (∼5% v/v ethanol)**				
**Fermentation**	Water	23 L	$0.00	Cost negligible
	Extract can	1.7 kg	$10.99	Coopers Home Brew Lager 1.7 kg (Dan Murphy’s)
	Fermentable sugar	1 kg	$3.89	Coopers Brew Enhancer No. 1 1 kg (Dan Murphy’s)
	Yeast		$0.00	Included with Extract can
**Bottling**	Carbonation drops	184 g	$1.91	8 g/L using Coopers Carbonation Drops 250 g (Dan Murphy’s)
	Bottles		$0.00	Assume collected not bought
		**Cost per 23 L**	**$16.79**	
		**Cost per 375 mL equivalent**	**$0.27**	
**XXXX (4.6% v/v ethanol)**				
		**Cost per 23 L equivalent**	**$100.16**	XXXX Bitter Cans 30 block (Dan Murphy’s)
		**Cost per 375 mL can**	**$1.63**	
**VB (4.9% v/v ethanol)**				
		**Cost per 23 L equivalent**	**$98.03**	Victoria Bitter Cans 30 Block (Dan Murphy’s)
		**Cost per 375 mL can**	**$1.60**	

As our data show that home brewed “Vegemite Beer” could be easily made from sugar, Vegemite, and yeast, we estimated the real-world cost of this product. Concentrations as low as 0.5% v/v of Vegemite or Marmite were sufficient to enable efficient and complete fermentation (Figs. 2A and 2C). Higher concentrations of Vegemite or Marmite could be used if a more Stout-like appearance was desired (Fig. 1), with a somewhat increased cost. We estimated the ingredients costs for batches of “Vegemite Beer,” standard home brewed beer made with a commercial retail kit, and equivalent retail-purchased volumes of the popular Australian beers XXXX and VB (Table 5). Retail cost of XXXX and VB purchased in bulk is approximately $1.60 per 375 mL. Ingredients costs for an equivalent volume of home brewed beer made with a commercial home brew kit is ∼$0.27. The estimated costs of “Vegemite Beer” is ∼$0.09 per 375 mL. “Vegemite Beer” is therefore substantially cheaper than other readily available products.

## Conclusion

We show that Vegemite, Marmite, or similar yeast extract spreads can be added at low levels to glucose solutions to provide a complete nutrient source for additionally inoculated yeast to efficiently grow and ferment. This has implications for control of illicit alcohol production.

##  Supplemental Information

10.7717/peerj.2271/supp-1 Supplemental Information 1 Headspace Gas Chromatography

## Additional Information and Declarations

### Data Availability

The following information was supplied regarding data availability: PRIDE ProteomeXchange title: Vegemite Beer ProteomeXchange accession: PXD003402
Project Webpage: http://www.ebi.ac.uk/pride/archive/projects/PXD003402
FTP Download: ftp://ftp.pride.ebi.ac.uk/pride/data/archive/2016/08/PXD003402.
