# Supplementary material for: Vegemite Beer: yeast extract spreads as nutrient supplements to promote fermentation
Source: PeerJ. 2016 Aug 10;4:e2271. doi: 10.7717/peerj.2271 (PMC4991886; doi:10.7717/peerj.2271)
Supplement: Supplemental Information 1 [file peerj-04-2271-s001.zip › Vegemite 100915 006.pdf]

| | | | | | |
|---|---|---|---|---|---|
| Software Version | : | 6.3.2.0646 | Date | : | 9/10/2015 11:18:50 AM |
| Reprocess Number | : | uqchem-gcms: 3918 | | | |
| Sample Name | : | | | | |
| Instrument Name | : | 680GC | Data Acquisition Time | : | 9/10/2015 11:16:08 AM |
| Rack/Vial | : | 0/0 | Channel | : | B |
| Sample Amount | : | 1.000000 | Operator | : | manager |
| Cycle | : | 6 | Dilution Factor | : | 1.000000 |

Result File : c:\users\uq chem\desktop\fid run tcws 6.3.2\data\Vegemite 100915 006.rst
Sequence File : C:\Users\UQ Chem\Desktop\FID RUN TCWS 6.3.2\Sequence\Beer ethanol HS.seq

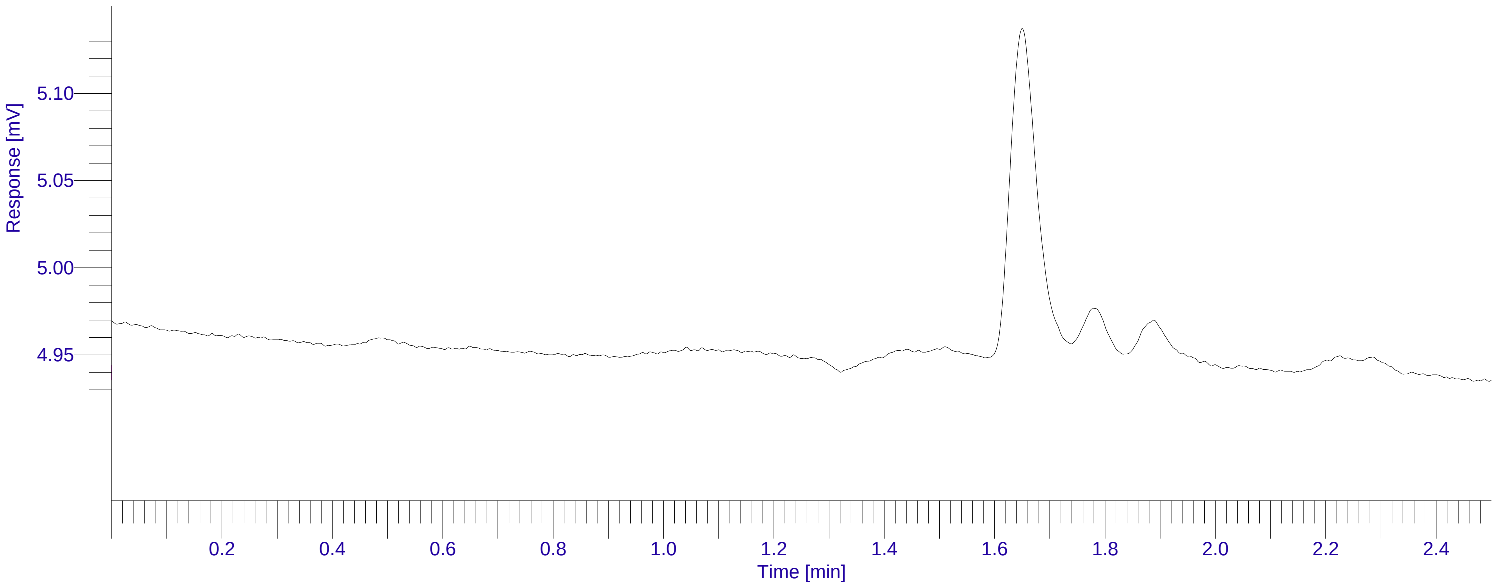


# DEFAULT REPORT

No peaks available to report
