# Supplementary material for: Vegemite Beer: yeast extract spreads as nutrient supplements to promote fermentation
Source: PeerJ. 2016 Aug 10;4:e2271. doi: 10.7717/peerj.2271 (PMC4991886; doi:10.7717/peerj.2271)
Supplement: Supplemental Information 1 [file peerj-04-2271-s001.zip › Vegemite 100915 010.pdf]

Software Version : 6.3.2.0646
Reprocess Number : uqchem-gcms: 3922
Sample Name :
Instrument Name : 680GC
Rack/Vial : 0/0
Sample Amount : 1.000000
Cycle : 10

Date : 9/10/2015 11:32:29 AM

Data Acquisition Time : 9/10/2015 11:29:48 AM
Channel : B
Operator : manager
Dilution Factor : 1.000000

Result File : c:\users\uq chem\desktop\fid run tcws 6.3.2\data\Vegemite 100915 010.rst
Sequence File : C:\Users\UQ Chem\Desktop\FID RUN TCWS 6.3.2\Sequence\Beer ethanol HS.seq

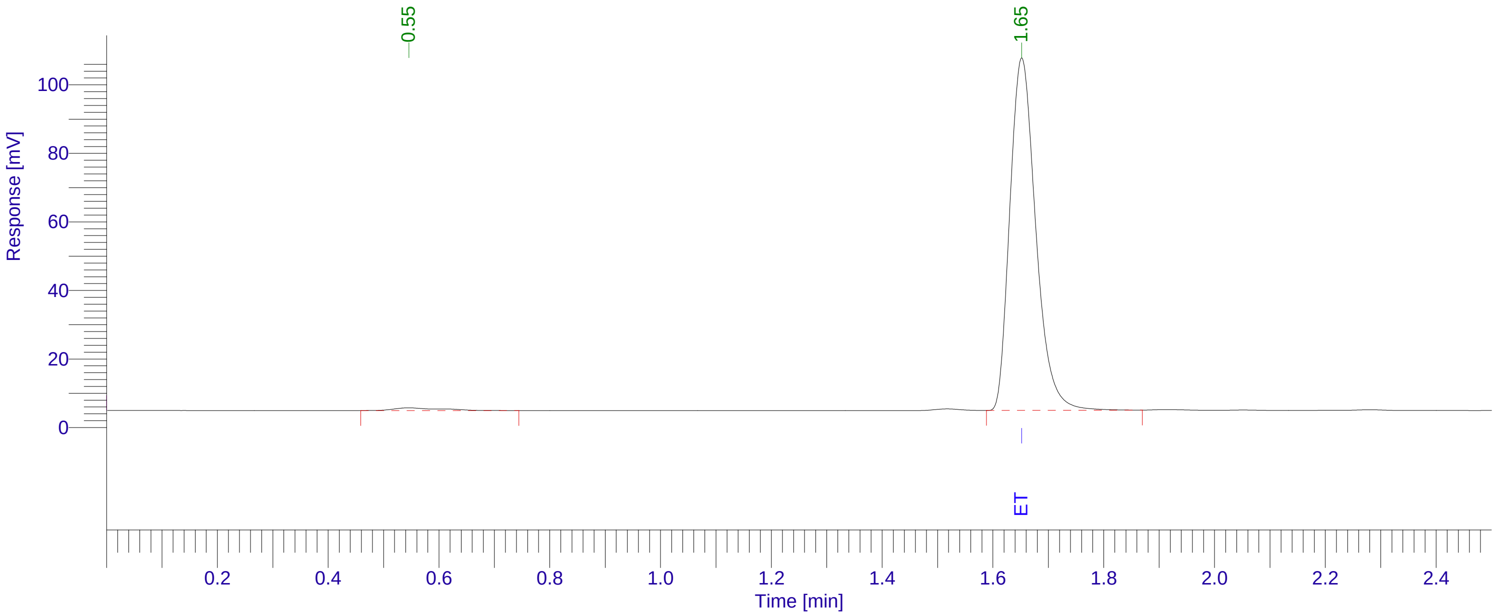

DEFAULT REPORT

Peak #	Component Name	Time [min]	Area [µV·s]	Height [µV]
1		0.546	4538.47	790.60
2	ETHANOL	1.652	334759.34	102904.92
			339297.82	103695.52
