# Supplementary material for: Vegemite Beer: yeast extract spreads as nutrient supplements to promote fermentation
Source: PeerJ. 2016 Aug 10;4:e2271. doi: 10.7717/peerj.2271 (PMC4991886; doi:10.7717/peerj.2271)
Supplement: Supplemental Information 1 [file peerj-04-2271-s001.zip › Vegemite 100915 016.pdf]

| | | | | |
|---|---|---|---|---|
| Software Version | : 6.3.2.0646 | | Date | : 9/10/2015 11:53:00 AM |
| Reprocess Number | : uqchem-gcms: 3928 | | | |
| Sample Name | : | | | |
| Instrument Name | : 680GC | | Data Acquisition Time | : 9/10/2015 11:50:17 AM |
| Rack/Vial | : 0/0 | | Channel | : B |
| Sample Amount | : 1.000000 | | Operator | : manager |
| Cycle | : 16 | | Dilution Factor | : 1.000000 |

Result File : c:\users\uq chem\desktop\fid run tcws 6.3.2\data\Vegemite 100915 016.rst
Sequence File : C:\Users\UQ Chem\Desktop\FID RUN TCWS 6.3.2\Sequence\Beer ethanol HS.seq

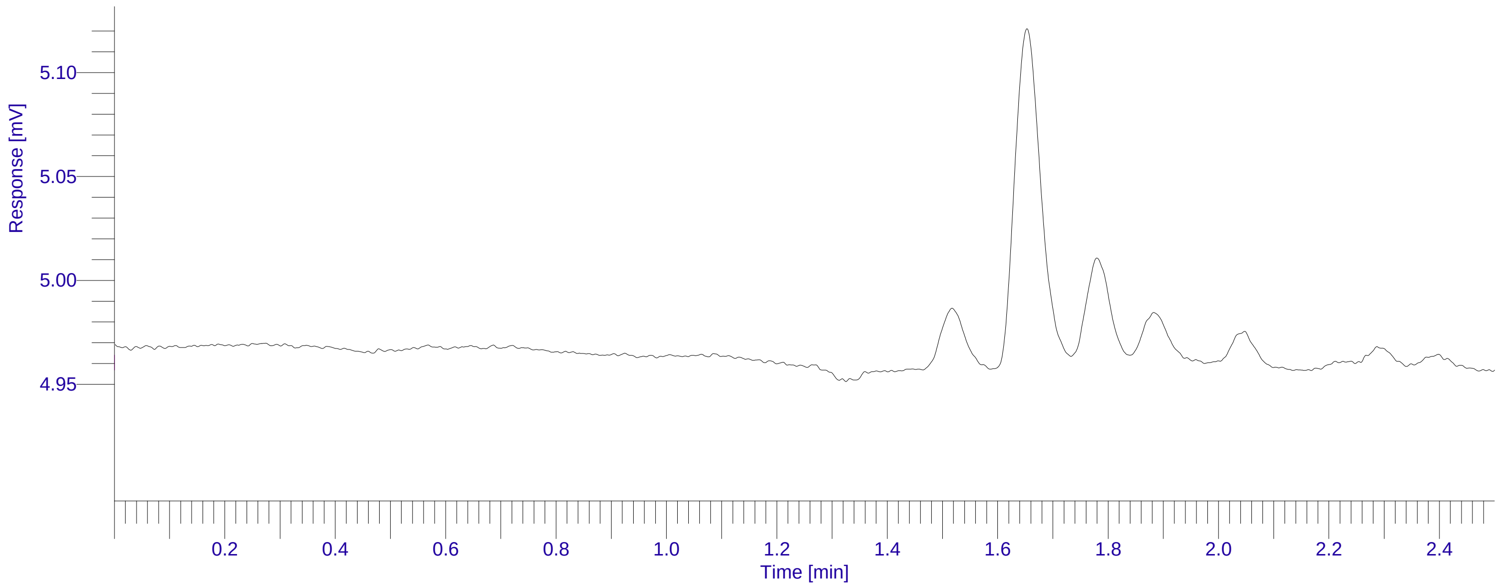


# DEFAULT REPORT

No peaks available to report
