# Supplementary material for: Vegemite Beer: yeast extract spreads as nutrient supplements to promote fermentation
Source: PeerJ. 2016 Aug 10;4:e2271. doi: 10.7717/peerj.2271 (PMC4991886; doi:10.7717/peerj.2271)
Supplement: Supplemental Information 1 [file peerj-04-2271-s001.zip › Vegemite 100915 021.pdf]

Software Version : 6.3.2.0646
Reprocess Number : uqchem-gcms: 3933
Sample Name :
Instrument Name : 680GC
Rack/Vial : 0/0
Sample Amount : 1.000000
Cycle : 21

Date : 9/10/2015 12:11:05 PM

Data Acquisition Time : 9/10/2015 12:08:22 PM
Channel : B
Operator : manager
Dilution Factor : 1.000000

Result File : c:\users\uq chem\desktop\fid run tcws 6.3.2\data\Vegemite 100915 021.rst
Sequence File : C:\Users\UQ Chem\Desktop\FID RUN TCWS 6.3.2\Sequence\Beer ethanol HS.seq

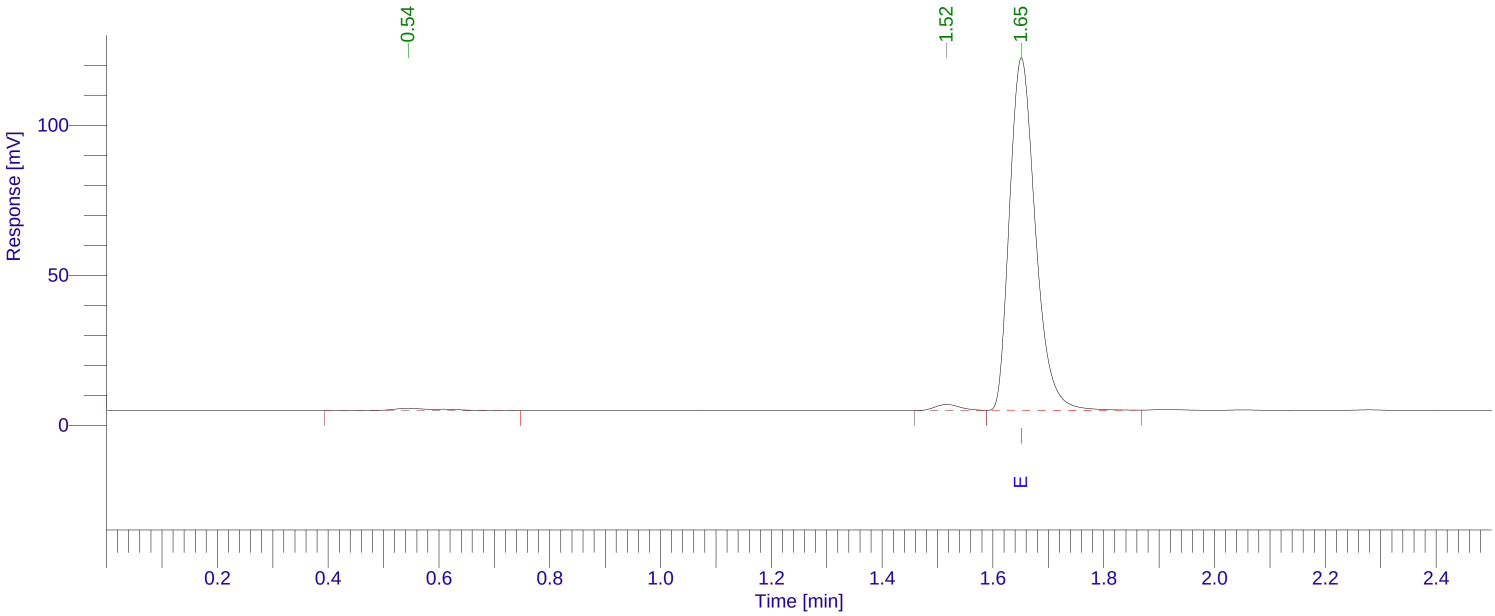

DEFAULT REPORT

Peak #	Component Name	Time [min]	Area [µV·s]	Height [µV]
1		0.545	4319.19	760.39
2		1.516	6381.81	2014.68
3	ETHANOL	1.651	380756.58	117471.88
			391457.58	120246.95
