# Supplementary material for: Vegemite Beer: yeast extract spreads as nutrient supplements to promote fermentation
Source: PeerJ. 2016 Aug 10;4:e2271. doi: 10.7717/peerj.2271 (PMC4991886; doi:10.7717/peerj.2271)
Supplement: Supplemental Information 1 [file peerj-04-2271-s001.zip › Vegemite 100915 025.pdf]

Software Version	: 6.3.2.0646	Date	: 9/10/2015 12:24:50 PM
Reprocess Number	: uqchem-gcms: 3937		
Sample Name	:		
Instrument Name	: 680GC	Data Acquisition Time	: 9/10/2015 12:22:02 PM
Rack/Vial	: 0/0	Channel	: B
Sample Amount	: 1.000000	Operator	: manager
Cycle	: 25	Dilution Factor	: 1.000000

Result File : c:\users\uq chem\desktop\fid run tcws 6.3.2\data\Vegemite 100915 025.rst
Sequence File : C:\Users\UQ Chem\Desktop\FID RUN TCWS 6.3.2\Sequence\Beer ethanol HS.seq

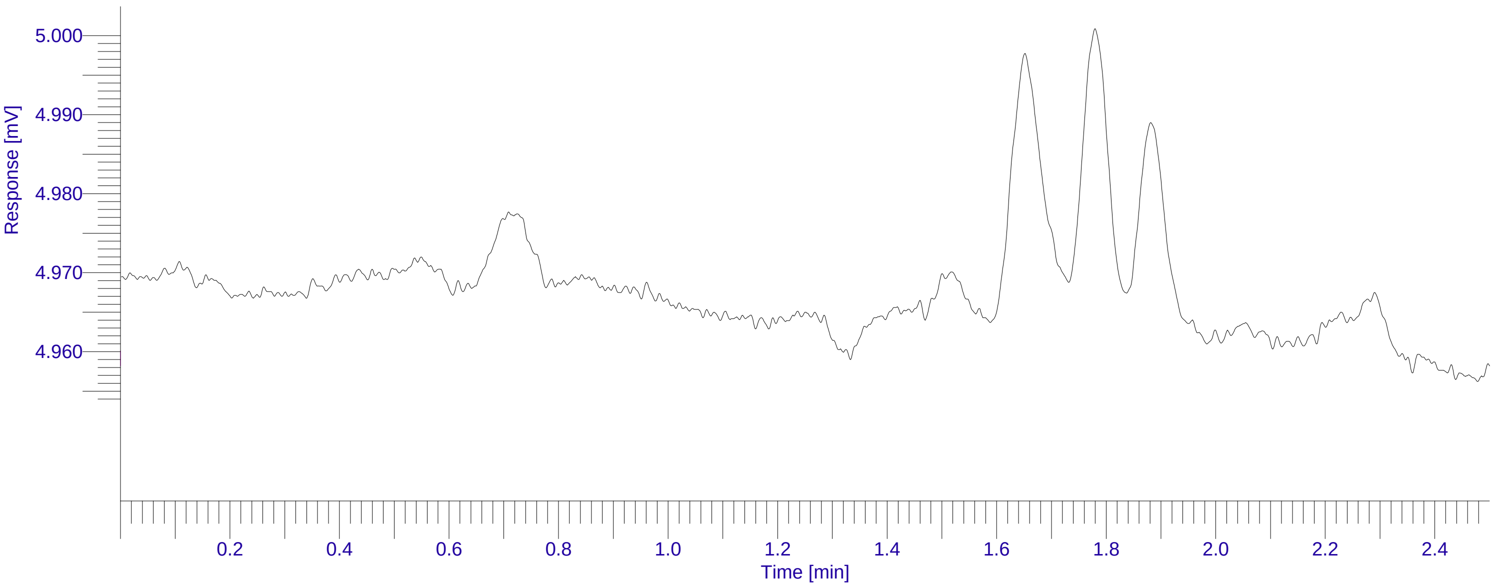

DEFAULT REPORT

No peaks available to report
