# Supplementary material for: Vegemite Beer: yeast extract spreads as nutrient supplements to promote fermentation
Source: PeerJ. 2016 Aug 10;4:e2271. doi: 10.7717/peerj.2271 (PMC4991886; doi:10.7717/peerj.2271)
Supplement: Supplemental Information 1 [file peerj-04-2271-s001.zip › Vegemite 100915 029.pdf]

Software Version	:	6.3.2.0646	Date	:	9/10/2015 12:38:26 PM
Reprocess Number	:	uqchem-gcms: 3941			
Sample Name	:		Data Acquisition Time	:	9/10/2015 12:35:42 PM
Instrument Name	:	680GC	Channel	:	B
Rack/Vial	:	0/0	Operator	:	manager
Sample Amount	:	1.000000	Dilution Factor	:	1.000000
Cycle	:	29			

Result File : c:\users\uq chem\desktop\fid run tcws 6.3.2\data\Vegemite 100915 029.rst
Sequence File : C:\Users\UQ Chem\Desktop\FID RUN TCWS 6.3.2\Sequence\Beer ethanol HS.seq

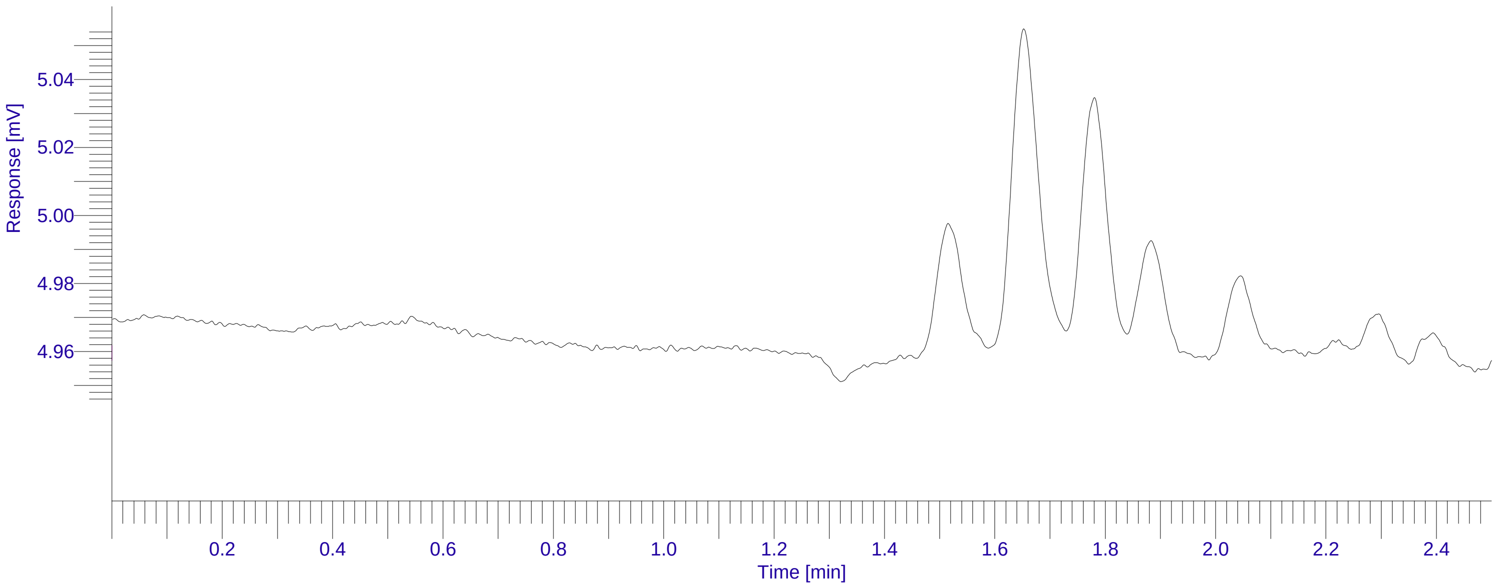

DEFAULT REPORT

No peaks available to report
