# Supplementary material for: Vegemite Beer: yeast extract spreads as nutrient supplements to promote fermentation
Source: PeerJ. 2016 Aug 10;4:e2271. doi: 10.7717/peerj.2271 (PMC4991886; doi:10.7717/peerj.2271)
Supplement: Supplemental Information 1 [file peerj-04-2271-s001.zip › Vegemite 100915 formaldehyde.pdf]

Software Version	:	6.3.2.0646	Date	:	9/10/2015 12:45:45 PM
Reprocess Number	:	uqchem-gcms: 3942			
Sample Name	:		Data Acquisition Time	:	9/10/2015 12:43:05 PM
Instrument Name	:	680GC	Channel	:	B
Rack/Vial	:	0/0	Operator	:	manager
Sample Amount	:	1.000000	Dilution Factor	:	1.000000
Cycle	:	1			

Result File : c:\users\uq chem\desktop\fid run tcws 6.3.2\data\Vegemite 100915 formaldehyde.rst
Sequence File : C:\Users\UQ Chem\Desktop\FID RUN TCWS 6.3.2\Sequence\Beer ethanol HS_2.seq

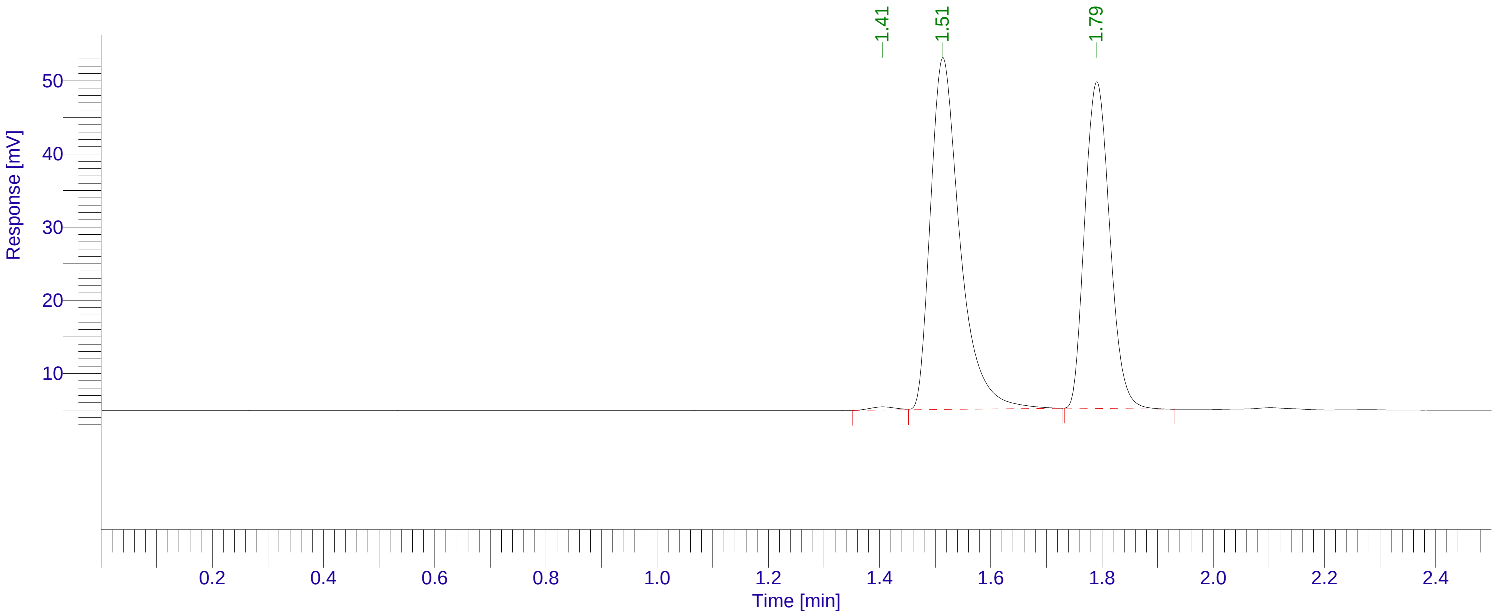

DEFAULT REPORT

Peak #	Component Name	Time [min]	Area [µV·s]	Height [µV]
1		1.405	1315.73	426.58
2		1.514	177112.48	48096.65
3		1.790	139524.15	44666.85
			317952.36	93190.08
