# Supplementary material for: Vegemite Beer: yeast extract spreads as nutrient supplements to promote fermentation
Source: PeerJ. 2016 Aug 10;4:e2271. doi: 10.7717/peerj.2271 (PMC4991886; doi:10.7717/peerj.2271)
Supplement: Supplemental Information 1 [file peerj-04-2271-s001.zip › Vegemite 100915 MeOH.pdf]

Software Version	: 6.3.2.0646		Date	: 9/10/2015 12:52:27 PM
Reprocess Number	: uqchem-gcms: 3944			
Sample Name	:			
Instrument Name	: 680GC		Data Acquisition Time	: 9/10/2015 12:49:44 PM
Rack/Vial	: 0/0		Channel	: B
Sample Amount	: 1.000000		Operator	: manager
Cycle	: 3		Dilution Factor	: 1.000000

Result File : c:\users\uq chem\desktop\fid run tcws 6.3.2\data\Vegemite 100915 MeOH.rst
Sequence File : C:\Users\UQ Chem\Desktop\FID RUN TCWS 6.3.2\Sequence\Beer ethanol HS_2.seq

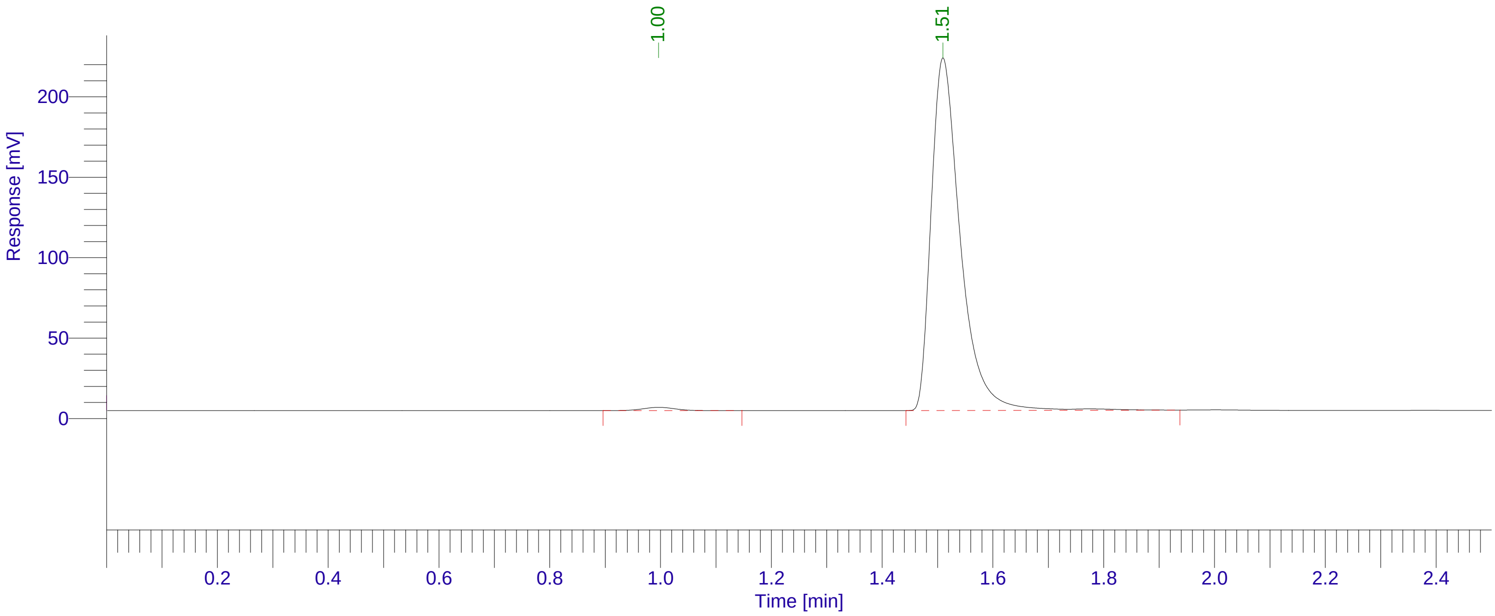

DEFAULT REPORT

Peak #	Component Name	Time [min]	Area [µV·s]	Height [µV]
1		0.996	7882.85	1995.93
2		1.510	803607.74	219288.85
			811490.59	221284.78
